# Association Between Anti-CD20 Therapies and COVID-19 Severity Among Patients With Relapsing-Remitting and Progressive Multiple Sclerosis

**DOI:** 10.1001/jamanetworkopen.2023.19766

**Published:** 2023-06-23

**Authors:** Edouard Januel, David Hajage, Pierre Labauge, Elisabeth Maillart, Jérome De Sèze, Hélène Zephir, Jean Pelletier, Laurent Guilloton, Caroline Bensa, Olivier Heinzlef, Olivier Casez, Damien Biotti, Bertrand Bourre, Sandra Vukusic, Aude Maurousset, Eric Berger, David Laplaud, Christine Lebrun-Frénay, Anne-Laure Dubessy, Pierre Branger, Eric Thouvenot, Pierre Clavelou, François Sellal, Eric Manchon, Thibault Moreau, Caroline Papeix, Florence Tubach, Céline Louapre

**Affiliations:** 1 Sorbonne Université, INSERM, Institut Pierre Louis d’Epidémiologie et de Santé Publique, Assistance Publique Hôpitaux de Paris, Hôpital Pitié Salpêtrière, Département de Santé Publique, Centre de Pharmacoépidémiologie (Cephepi), Unité de Recherche Clinique PSL-CFX, Paris, France; 2 Sorbonne Université, Assistance Publique des Hôpitaux de Paris, Hôpital de la Pitié Salpêtrière, Institut National de la Santé et de la Recherche Médicale, Centre National de la Recherche Scientifique, Neuroscience Clinical Investigation Center, Paris Brain Institute, Paris, France; 3 Department of Neurology, CRC-SEP, Montpellier University Hospital, Montpellier, France/Institute for Neurosciences of Montpellier, INSERM and University of Montpellier, Montpellier, France; 4 Department of Neurology and Clinical Investigation Center, CHU de Strasbourg, CIC 1434, INSERM 1434, Strasbourg, France; 5 Department of Neurology, CHU Lille, INSERM U1172, University of Lille, Lille, France; 6 Aix Marseille University, Assistance Publique Hopitaux de Marseille, Hôpital de la Timone, Pôle de Neurosciences Cliniques, Service de Neurologie, Marseille, France; 7 Association des Neurologues Libéraux de Langue Française, Bergerac, France; 8 Département de Neurologie, Hôpital Fondation Adolphe de Rothschild, Paris, France; 9 Département de Neurologie, CRC-SEP, Centre Hospitalier de Poissy-St Germain-en-Laye, France; 10 Neurologie, Pathologies Inflammatoires du Système Nerveux, CHU Grenoble Alpes, Grenoble, France / Techniques de l’Ingénierie Médicale et de la Complexité–Informatique, Mathématiques et Applications, Grenoble, Translational Research in Autoimmunity and Inflammation Group, Université de Grenoble Alpes, Grenoble, France; 11 Centre Ressources et Compétences Sclérose en Plaques (CRC-SEP) et Service de Neurologie B4, Hôpital Pierre-Paul Riquet, CHU Toulouse Purpan, Toulouse, France INSERM UMR1291–CNRS UMR5051, Institut Toulousain des Maladies Infectieuses et Inflammatoires (Infinity), Université Toulouse 3, Toulouse, France; 12 Department of Neurology, CHU Rouen, Rouen, France; 13 Hospices Civils de Lyon, Hôpital Neurologique, Service de Neurologie, Sclérose en Plaques, Pathologies de la Myéline et Neuro-Inflammation, Bron, France; 14 CRC-SEP and Department of Neurology, CHU de Tours, Hôpital Bretonneau, Tours, France; 15 CHU de Besançon, Service de Neurologie, Besançon, France; 16 CHU de Nantes, Service de Neurologie & CIC015 INSERM, Nantes, France; 17 CRC-SEP CHU Nice, UR2CA-URRIS, Université Nice Cote d’Azur, Hôpital Pasteur 2, Nice, France; 18 Assistance Publique Hôpitaux de Paris, Sorbonne Université, Department of Neurology, Saint-Antoine Hospital, CRC-SEP Paris, Paris, France; 19 Service de Neurologie, CHU de Caen Normandie, Caen, France; 20 Department of Neurology, Nimes University Hospital, Nimes, France; 21 Department of Neurology, CHU Clermont-Ferrand, Clermont-Ferrand, France; 22 Département de Neurologie, Hôpitaux Civils de Colmar, Unité INSERM U-1118, Faculté de Médecine, Université de Strasbourg, Strasbourg, France; 23 Department of Neurology, Gonesse Hospital, Gonesse, France; 24 Department of Neurology, CHU de Dijon, Dijon, France; 25 Institut de Génomique Fonctionnelle, UMR5203, INSERM 1191, Université de Montpellier, Montpellier, France

## Abstract

**Importance:**

In patients with multiple sclerosis (MS), factors associated with severe COVID-19 include anti-CD20 therapies and neurologic disability, but it is still unclear whether these 2 variables are independently associated with severe COVID-19 or whether the association depends on MS clinical course.

**Objective:**

To assess the association between anti-CD20 therapies and COVID-19 severity in patients with relapsing-remitting MS (RRMS) and progressive MS (PMS).

**Design, Setting, and Participants:**

This multicenter, retrospective cohort study used data from the COVISEP study, which included patients with MS and COVID-19 from February 1, 2020, to June 30, 2022, at 46 French MS expert centers, general hospitals, and private neurology practices. Eligible patients with RRMS were those treated with high-efficacy MS therapy (ie, anti-CD20, fingolimod, or natalizumab), and eligible patients with PMS were those younger than 70 years with an Expanded Disability Status Scale (EDSS) score of 8 or lower. Patients were monitored from COVID-19 symptom onset until recovery or death.

**Exposures:**

Current anti-CD20 therapy (ocrelizumab or rituximab).

**Main Outcomes and Measures:**

The main outcome was severe COVID-19 (ie, hospitalization with any mode of oxygenation or death). All analyses were conducted separately in patients with RRMS and PMS using propensity score–weighted logistic regression. Subgroup analyses were performed according to COVID-19 vaccine status, sex, EDSS score, and age.

**Results:**

A total of 1400 patients, 971 with RRMS (median age, 39.14 years [IQR, 31.38-46.80 years]; 737 [76.1%] female) and 429 with PMS (median age, 54.21 years [IQR, 48.42-60.14 years]; 250 [58.3%] female) were included in the study. A total of 418 patients with RRMS (43.0%) and 226 with PMS (52.7%) were treated with anti-CD20 therapies. In weighted analysis, 13.4% and 2.9% of patients with RRMS treated and not treated with anti-CD20 had severe COVID-19, respectively, and anti-CD20 treatment was associated with increased risk of severe COVID-19 (odds ratio [OR], 5.20; 95% CI, 2.78-9.71); this association persisted among vaccinated patients (7.0% vs 0.9%; OR, 8.85; 95% CI, 1.26-62.12). Among patients with PMS, 19.0% and 15.5% of patients treated and not treated with anti-CD20 had severe COVID-19, respectively, and there was no association between anti-CD20 treatment and severe COVID-19 (OR, 1.28; 95% CI, 0.76-2.16). In PMS subgroup analysis, anti-CD20 exposure interacted negatively with EDSS score (*P* = .009 for interaction) and age (*P* = .03 for interaction); anti-CD20 therapies were associated with risk of severe COVID-19 only in patients with less neurologic disability and younger patients with PMS.

**Conclusions and Relevance:**

In this cohort study, risk of severe COVID-19 was higher in patients with PMS than in those with RRMS. Use of anti-CD20 therapies was associated with an increased risk of severe COVID-19 among patients with RRMS. In patients with PMS, there was no association between anti-CD20 therapies and risk of severe COVID-19.

## Key Points

**Question:**

Does the association between anti-CD20 therapies and risk of severe COVID-19 differ based on multiple sclerosis (MS) clinical course: relapsing-remitting MS (RRMS) or progressive MS (PMS)?

**Findings:**

In this cohort study of 1400 patients with MS and COVID-19, in patients with RRMS, anti-CD20 therapy was associated with an increased risk of severe COVID-19. Patients with PMS had higher risk of severe COVID-19 than patients with RRMS, but anti-CD20 exposure was not associated with severe COVID-19 in this population.

**Meaning:**

In this study, patients with PMS had higher risk of severe COVID-19 than patients with RRMS, and anti-CD20 therapies were associated with severe COVID-19 in patients with RRMS.

## Teaser

This cohort study assesses the association of anti-CD20 therapies with risk of severe COVID-19 in a French cohort of patients with relapsing-remitting or progressive multiple sclerosis.

## Introduction

The COVID-19 pandemic led to more than 14 million deaths worldwide^1^ and still represents a threat for public health, particularly for patients with multiple sclerosis (MS). According to 1 report from Italy, the risk of hospitalization for COVID-19 among patients with MS was twice the risk in an age- and sex-matched population.^2^ As in the general population, age, male sex, and cardiovascular comorbidities are associated with severe outcome among patients with MS and COVID-19.^3,4^ Additionally, 2 MS-specific risk factors are associated with severe COVID-19: neurologic disability^3,4^ and anti-CD20 therapies.^4,5,6,7,8^ However, it is still unclear whether these 2 variables are independently associated with severe COVID-19 or whether the association depends on MS phenotype. Progressive MS (PMS), including primary or secondary PMS, is associated with a higher risk of severe infection.^9^ Patients with PMS also have a higher likelihood of being hospitalized for COVID-19 compared with those with relapsing-remitting MS (RRMS).^3^ Anti-CD20 therapies are unique MS disease-modifying therapies (DMTs) prescribed both for PMS and for RRMS,^10,11^ whereas all other DMTs are recommended for patients with RRMS only (some off-label DMTs are anecdotally used in patients with PMS).^12^ Previous studies^3,4,5,6,7,8^ assessing severe COVID-19 risk associated with anti-CD20 therapies in patients with MS mixed patients with RRMS and PMS and used varying reference groups (no DMTs, first-line therapies, or other high-efficacy therapies). Thus, it is unclear whether the association between anti-CD20 therapies and severe COVID-19 risk is partially driven by the underlying background (eg, age, neurologic disability, and comorbidity) of patients treated with anti-CD20 therapies or by the immunosuppressive effects associated with anti-CD20 therapies. Due to the considerable demographic and neurologic disability differences between MS subgroups, the association of anti-CD20 therapies with COVID-19 severity should be assessed separately among patients with RRMS and PMS. Moreover, in RRMS, until the past few years, anti-CD20 therapies were usually proposed after failure of first-line therapies or for patients with more severe disease at diagnosis^13^; thus, in patients with RRMS, the association of anti-CD20 therapies with COVID-19 severity should be assessed only in those eligible to receive this treatment—that is, those treated with other high-efficacy DMTs.

The primary objective of this study was to assess the association of anti-CD20 therapies with risk of severe COVID-19 separately in patients with RRMS and PMS. Our secondary objectives were to evaluate whether the association of anti-CD20 therapies with COVID-19 severity was influenced by age, sex, neurologic disability (Expanded Disability Status Scale [EDSS]), and COVID-19 vaccination status and to evaluate the factors associated with severe COVID-19 among anti-CD20–treated patients with RRMS and PMS.

## Methods

### Design

This multicenter, retrospective cohort study used data from the COVISEP study, which included patients with MS and confirmed or highly suspected COVID-19. The current study received approval from the ethics committee of Sorbonne University. Patients included were informed about the objective of the study, and the collection of nonopposition to the use of medical data was carried out according to French law, good clinical practice, and the General Data Protection Regulation. Patients were informed that data collected in medical records might be used for research in accordance with privacy rules. The study followed the Strengthening Reporting of Observational Studies in Epidemiology (STROBE) reporting guideline.

### Study Population

Inclusion criteria were (1) MS; (2) biologically confirmed or highly suspected COVID-19 from February 1, 2020, to June 30, 2022; and (3) patient corresponding to the profile of use of anti-CD20 therapies in France—that is, for patients with RRMS, treatment with high-efficacy DMTs (in France, anti-CD20 [ocrelizumab, rituximab], fingolimod, or natalizumab), and for patients with PMS, age younger than 70 years and an EDSS score of 8 or less. Patients with PMS who were older than 70 years or had an EDSS score greater than 8 were not included because they do not correspond to the usual prescription of anti-CD20 in France; thus, including them would impair the comparability between anti-CD20–treated and anti-CD20–untreated patients with PMS. Exclusion criteria were COVID-19 reinfection, a lack of information concerning MS course or DMTs, the patient’s opposition to the use of their medical data, and for PMS, patients treated with unconventional drugs that have not been thoroughly evaluated or theoretically may be associated with increased risk of severe COVID-19 in patients with MS because they are immunosuppressive therapies (cyclophosphamide, alemtuzumab, mycophenolate mofetil, azathioprine, and methotrexate).

### Data Collection

Forty-six centers, consisting of all French MS expert centers, general hospitals, and private neurology practices, participated in patient inclusion between March 1, 2020, and June 30, 2022; details are described elsewhere.^3^ Recorded data are detailed in eMethods 1 in Supplement 1. Vaccination before COVID-19 was defined as SARS-CoV-2 infection occurring at least 7 days after the second dose of COVID-19 vaccine. COVID-19 severity was categorized on an ordinal scale ranging from 1 to 7: 1, no limitations on activities; 2, limitations without hospitalization; 3, hospitalization without supplemental oxygen; 4, hospitalization with supplemental oxygen; 5, hospitalization with noninvasive or high-flow oxygen devices; 6, hospitalization with invasive mechanical ventilation; and 7, death.^14^ The COVID-19 variant was proxied at the date of infection by the French COVID-19 variant surveillance, considering the variant dominant when it represented more than 50% of positive samples in the week of interest: original Alpha, before June 30, 2021; Delta, between June 30 and December 27, 2021; and Omicron, after December 27, 2021. Data were recorded by the treating neurologist at the subsequent consultation following COVID-19 resolution or at COVID-19–related hospitalization for patients with severe COVID-19.

### Outcomes

The primary outcome was severe COVID-19, defined by a severity score of 4 or greater (ie, hospitalization with any mode of oxygenation or death). This outcome was unlikely to be influenced by factors such as bed availability or surveillance for transient neurologic deterioration contemporary with COVID-19 infection or by principle for anti-CD20–treated patients with COVID-19. Secondary outcomes were a COVID-19 severity score of 3 or greater (minimum of hospitalization), 5 or greater (minimum of hospitalization with high-flow oxygen), or 7 (death).

### Statistical Analysis

All analyses were conducted separately in patients with RRMS and PMS. Categorical variables were compared by the χ^2^ or Fisher exact test and continuous variables by the *t* test or Wilcoxon rank sum test, as appropriate. The risk of severe COVID-19 (severity score ≥4) was compared between patients treated or not treated with anti-CD20 therapies using a propensity score framework. The propensity score was estimated with multivariable logistic regression including the following independent variables: age, sex, time from MS onset, EDSS score, comorbidities (cardiovascular disease, pulmonary disease, diabetes, obesity, and active tobacco smoking), high-dose methylprednisolone therapy in the month before COVID-19, postvaccine COVID-19, COVID-19 variant, and treatment of COVID-19 with monoclonal antibody. Then, a propensity score–weighted analysis was performed using inverse propensity score weights and the entire data set to estimate the average anti-CD20 effect in the overall population. Covariate balance between the 2 groups was assessed before and after weighting, and we considered an absolute standardized difference (ASD) less than 0.1 as evidence of balance. The association of anti-CD20 therapy with each of the outcomes was assessed using weighted logistic regression models. The SE of the estimated odds ratios (ORs) was estimated using a robust SE estimator. Planned subgroup analyses were performed according to age (divided at the median for RRMS and PMS), sex, EDSS score (<6 or ≥6), and COVID-19 vaccine status. No adjustment for multiple comparisons was done.

Factors associated with severe COVID-19 among anti-CD20–treated patients were evaluated using univariable and multivariable logistic regression models. Age and EDSS score (previously recognized as the main factors associated with severe COVID-19^3,4^) and all other variables associated with the outcome in the univariable analysis were included in the multivariable analysis.

Missing data were handled by multiple imputations (details are given in eMethods 2 in Supplement 1). Outcomes were included in the imputation model and imputed if missing.

Two-sided *P* < .05 defined statistical significance. All analyses were conducted using R, version 4.1.1 (R Project for Statistical Computing).

## Results

Of the 2700 patients included in the COVISEP cohort, 1400 were included in the analysis. A total of 971 met the inclusion criteria for RRMS (median age, 39.14 years [IQR, 31.38-46.80 years]; 737 [76.1%] female; 232 [23.9%] male; 2 [0.2%] without sex data) and 429 for PMS (median age, 54.21 years [IQR, 48.42-60.14 years]; 250 [58.3%] female; 179 [41.7%] male) (flowchart is given in eFigure 1 in Supplement 1).

### Patients With RRMS

Of the 971 patients with RRMS, 418 (43.0%) were treated with anti-CD20 (350 [83.7%] with ocrelizumab and 68 [16.3%] with rituximab) (eTable 1 in Supplement 1) and 553 (57.0%) with other high-efficacy DMTs (282 [51.0%] fingolimod and 271 [49.0%] natalizumab). Table 1 shows the characteristics of patients with RRMS before and after propensity score weighting. Among included patients with RRMS, those treated with anti-CD20 had a higher median EDSS score (2.00 [IQR, 1.00-4.00] vs 2.00 [IQR, 1.00-3.00]; *P* < .001) and a shorter median time from MS onset (8.96 years [IQR, 4.70-14.78 years] vs 10.37 years [IQR, 5.40-16.44 years]; *P* = .02). They were also more likely to have been vaccinated before having COVID-19 (169 of 418 [40.4%] vs 152 of 553 [27.5%]; *P* < .001) and to have been infected with the Delta or Omicron variant. Seventeen patients with RRMS treated with anti-CD20 (4.1%) vs 1 not treated with anti-CD20 (0.2%) were treated with antispike monoclonal antibody (*P* < .001). After propensity score weighting, all variables were well balanced (ASD <0.1).

**Table 1.** Demographic and Clinical Characteristics at the Date of SARS-CoV-2 Infection in Patients With RRMS and in the Propensity Score–Weighted Population According to MS Treatment

Characteristic	Total patients with RRMS (N = 971)^a^	Patients with RRMS before propensity score weighting			Propensity score–weighted population with RRMS		
		Treated with natalizumab or fingolimod (n = 553)^a^	Treated with anti-CD20 (n = 418)^a^	*P* value	Treated with natalizumab or fingolimod^b^	Treated with anti-CD20^b^	ASD
Sex^c^							
Female	737/969 (76.1)	411/552 (74.5)	326/417 (78.2)	.21	76.2	75.1	0.0112
Male	232/969 (23.9)	141/552 (25.5)	91/417 (21.8)		23.8	24.9	
Age, median (IQR), y^d^	39.14 (31.38-46.80)	39.14 (31.52-48.09)	39.15 (31.22-45.81)	.49	38.73 (31.3-47.72)	39.3 (31.23-46)	0.0091
EDSS score, median (IQR)^e^	2.00 (1.00-3.50)	2.00 (1.00-3.00)	2.00 (1.00-4.00)	<.001	1.76 (0.61-3.11)	1.71 (0.70-3.22)	0.0073
Time since MS onset, median (IQR), y^f^	9.76 (5.00-15.79)	10.37 (5.40-16.44)	8.96 (4.70-14.78)	.02	10.1 (4.97-16.01)	9.53 (4.90-15.6)	0.0046
Comorbidities							
Cardiovascular disease	28/971 (2.9)	17/553 (3.1)	11/418 (2.6)	.83	2.9	3.1	0.0024
Diabetes	12/971 (1.2)	5/553 (0.9)	7/418 (1.7)	.38	1.3	1.2	0.0007
Obesity	46/971 (4.7)	27/553 (4.9)	19/418 (4.5)	.93	4.9	5.0	0.0012
Pulmonary disease	27/971 (2.8)	15/553 (2.7)	12/418 (2.9)	>.99	2.7	2.7	0.0001
Smoking	98/971 (10.1)	56/553 (10.1)	42/418 (10.0)	>.99	10.2	10.3	0.0005
High-dose methylprednisolone during the month before COVID-19^g^	6/970 (0.6)	2/553 (0.4)	4/417 (1.0)	.41	0.6	0.6	0.0003
Vaccinated against COVID-19^h^	321/971 (33.1)	152/553 (27.5)	169/418 (40.4)	<.001	33.5	32.7	0.0073
SARS-CoV-2 variant^i^							
Original Alpha	565/969 (58.3)	354/551 (64.2)	211/418 (50.5)	<.001	58.3	58.9	0.0056
Delta	140/969 (14.4)	70/551 (12.7)	70/418 (16.7)		15.2	14.1	0.0111
Omicron	264/969 (27.2)	127/551 (23.0)	137/418 (32.8)		26.5	27.0	0.0056
Anti–COVID-19 monoclonal antibody treatment^j^	18/971 (1.9)	1/553 (0.2)	17/418 (4.1)	<.001	1.7	1.8	0.0013
Anti-CD20 treatment							
Ocrelizumab	NA	NA	350/418 (83.7)	NA	NA	NA	NA
Rituximab	NA	NA	68/418 (16.3)	NA	NA	NA	NA
Cycles of anti-CD20 treatment, median (IQR), No.^k^	NA	NA	4.00 (2.00-5.00)	NA	NA	NA	NA
Time from last anti-CD20 cycle to COVID-19, median (IQR), mo^l^	NA	NA	3.35 (1.54-5.03)	NA	NA	NA	NA

Abbreviations: ASD, absolute standardized difference; EDSS, Expanded Disability Status Scale; MS, multiple sclerosis; NA, not applicable; RRMS, relapsing-remitting multiple sclerosis.

^a^ Data are presented as number/total number (percentage) of patients, unless otherwise specified.

^b^ Data are presented as percentage of patients, unless otherwise specified.

^c^ Sex data were missing for 2 patients (0.2%).

^d^ Data were missing for 5 patients overall, 2 receiving natalizumab or fingolimod, and 3 receiving anti-CD20 therapies.

^e^ Data were missing for 55 patients overall, 29 receiving natalizumab or fingolimod, and 26 receiving anti-CD20 therapies.

^f^ Data were missing for 18 patients overall, 7 receiving natalizumab or fingolimod, and 11 receiving anti-CD20 therapies.

^g^ A high dose was 500 mg per day or greater for at least 1 day.

^h^ Infection occurring at least 7 days after the second dose of COVID-19 vaccine.

^i^ The variant was considered dominant when it was present in more than 50% of the samples for a given week: original Alpha, before June 30, 2021; Delta, from June 30 to December 27, 2021; and Omicron, after December 27, 2021.

^j^ Treatment with COVID-19 monoclonal antibody in the 5 days after symptom onset.

^k^ Data were missing for 61 patients receiving anti-CD20 therapies.

^l^ Data were missing for 30 patients receiving anti-CD20 therapies.

### Association Between Anti-CD20 Therapies and Severe COVID-19 Among Patients With RRMS

**Figure 1.**
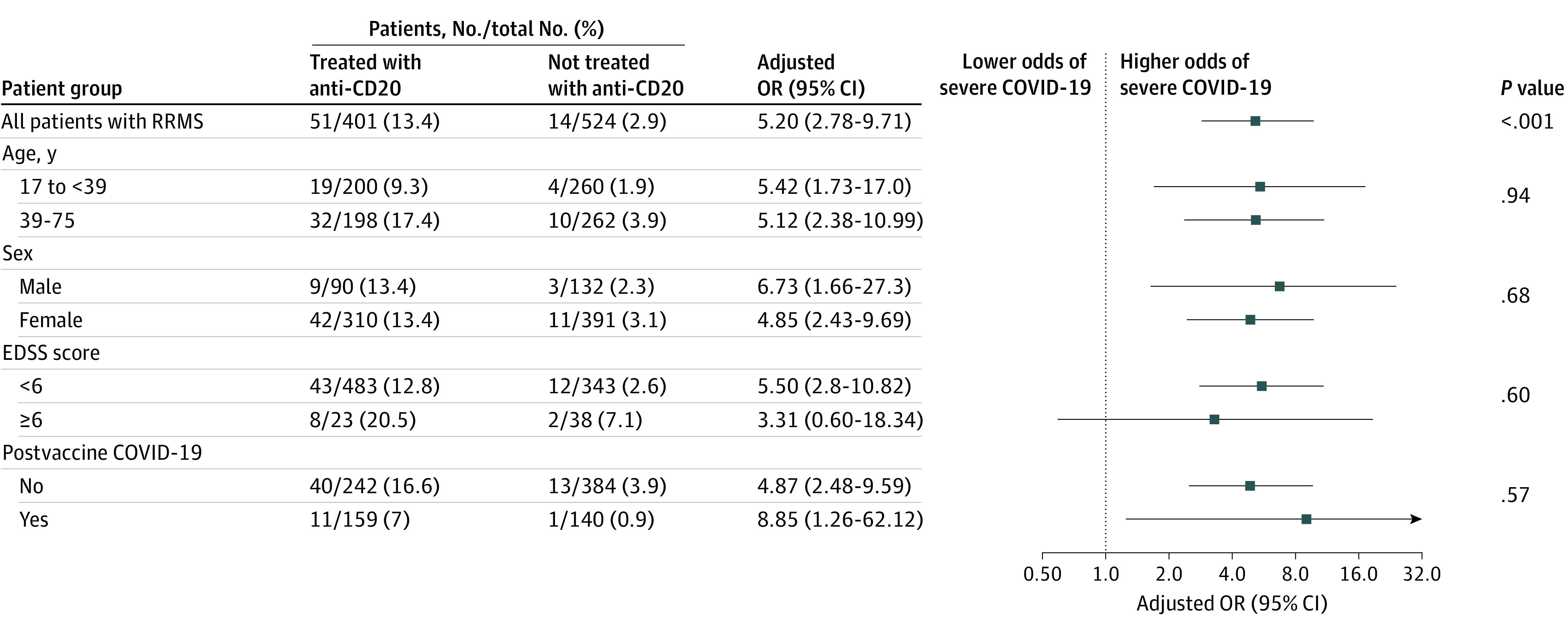
Association Between Anti-CD20 Therapies and Severe COVID-19 in All Patients With Relapsing-Remitting Multiple Sclerosis (RRMS) and by Subgroup Observed numbers of events are reported in the nonweighted population without imputation of missing data. Percentages and odds ratios (ORs) were estimated in the weighted population with imputation of missing data (including outcomes). The analysis was propensity score weighted (average treatment effect estimand). Variables used in the propensity score estimation were age, sex, time since multiple sclerosis onset, Expanded Disability Status Scale (EDSS) score, comorbidities (cardiovascular disease, pulmonary disease, diabetes, obesity, and active tobacco smoking), exposure to high-dose methylprednisolone in the month before COVID-19, postvaccine COVID-19 (infection occurring at least 7 days after the second dose of COVID-19 vaccine), previous COVID-19, COVID-19 variant (original Alpha, Delta, or Omicron), and treatment of COVID-19 with monoclonal antibody. Severe COVID-19 was defined as hospitalization with any mode of oxygenation or death. Age was divided into 2 groups based on the median for patients with RRMS, and subgroup analyses were conducted in age categories defined accordingly. *P* values for the subgroup analyses are for the interaction. Squares indicate ORs, with horizontal lines indicating 95% CIs.

Results of the analysis of the association between anti-CD20 therapies and severe COVID-19 in patients with RRMS are shown in Figure 1. Severe COVID-19 occurred in 51 patients (12.7%) treated with anti-CD20 vs 14 (2.7%) treated with natalizumab or fingolimod (nonweighted percentages); anti-CD20 treatment was associated with increased risk of severe COVID-19 (crude OR, 5.32; 95% CI, 2.89-9.81). In the propensity score–weighted analysis, 13.4% of anti-CD20–treated patients vs 2.9% in the untreated group had severe COVID-19, and anti-CD20 treatment was associated with increased risk of severe COVID-19 (adjusted OR, 5.20; 95% CI, 2.78-9.71). Subgroup crude and weighted analyses showed associations between anti-CD20 therapies and increased risk of severe COVID-19 regardless of age, sex, and vaccination status and in patients with an EDSS score less than 6 but not in patients with an EDSS score of 6 or higher. In weighted analysis, among unvaccinated patients with RRMS, 16.6% of anti-CD20–treated patients had severe COVID-19 in comparison with 3.9% not treated with anti-CD20. Among vaccinated patients with RRMS, 7.0% of anti-CD20–treated patients had severe COVID-19 in comparison with 0.9% not treated with anti-CD20, and treatment was associated with increased risk of severe COVID-19 (adjusted OR, 8.85; 95% CI, 1.26-62.12) (eTable 2 in Supplement 1). Considering COVID-19 secondary outcomes, in weighted analysis of patients with RRMS, 16.0% of those who were treated with anti-CD20 and 3.7% of those who were not had a severity score of 3 or greater, and treatment was associated with increased risk of severe COVID-19 (adjusted OR, 4.92; 95% CI, 2.83-8.56). Also, 4.5% of those who were treated with anti-CD20 and 0.8% of those who were not had a severity score of 5 or greater, and treatment was associated with increased risk of severe COVID-19 (adjusted OR, 5.72; 95% CI, 1.90-17.02). Among patients with RRMS, 2 (0.5%) treated with anti-CD20 died compared with none of the patients not treated with anti-CD20 (eTable 3 and eFigure 2 in Supplement 1).

### Factors Associated With Severe COVID-19 Among Patients With RRMS Treated With Anti-CD20

In univariable analysis, of 401 patients with RRMS who were treated with anti-CD20 and had an available outcome, the 51 who experienced severe COVID-19 (12.7%) were older, had more neurologic disability, had received more anti-CD20 infusions, and were more frequently treated with rituximab (eTable 4 in Supplement 1). Eleven of the 51 patients who had severe COVID-19 (21.6%) had been vaccinated (vs 148 of the 350 patients with nonsevere COVID-19 [42.3%]). The Omicron variant was less frequent among patients with severe COVID-19 (6 of 51 [11.8%]) vs nonsevere COVID-19 (124 of 350 [35.4%]). In multivariable analysis, none of the variables were significantly associated with COVID-19 severity.

### Patients With PMS

Of the 429 patients with PMS, 226 (52.7%) were treated with anti-CD20 therapies (104 [46.0%] with ocrelizumab and 122 [54.0%] with rituximab) (eTable 1 in Supplement 1) and 203 (47.3%) were not (150 [74.0%] received no treatment; 7 [3.4%], glatiramer acetate; 3 [1.4%], interferon; 10 [4.9%], dimethyl fumarate; 9 [4.4%], teriflunomide; 16 [7.9%], fingolimod; and 8 [3.9%], natalizumab). Characteristics of patients with PMS are reported in Table 2. Patients treated with anti-CD20 were younger (median age, 53.16 years [IQR, 46.84-58.62 years] vs 56.57 years [IQR, 50.94-61.60 years]; *P* < .001), had a shorter median time from MS onset (13.78 years [IQR, 8.83-21.75 years] vs 18.81 years [IQR, 11.28-27.23 years]; *P* < .001), were more frequently vaccinated before having COVID-19 (94 [41.6%] vs 32 [15.8%]; *P* < .001), and were more frequently infected with the Delta and Omicron variants compared with patients not treated with anti-CD20. Propensity score weighting allowed balancing of all variables between patients with PMS treated and not treated with anti-CD20 (ASD <0.1).

**Table 2.** Demographic and Clinical Characteristics at the Date of SARS-CoV-2 Infection in Patients With PMS and in the Propensity Score–Weighted Population According to MS Treatment

Characteristic	Total patients with PMS (N = 429)^a^	Patients with PMS before propensity score weighting			Propensity score–weighted population with PMS			
		Not treated with anti-CD20 (n = 203)^a^	Treated with anti-CD20 (n = 226)^a^	*P* value	Not treated with anti-CD20^b^	Treated with anti-CD20^b^	ASD
Sex							
Female	250 (58.3)	126 (62.1)	124 (54.9)	.16	55.9	57.6	0.0170
Male	179 (41.7)	77 (37.9)	102 (45.1)		40.1	32.4	
Age, median (IQR), y	54.21 (48.42-60.14)	56.57 (50.94-61.60)	53.16 (46.84-58.62)	<.001	53.56 (45.57-60.15)	54.02 (48.19-59.37)	0.0521
EDSS score, median (IQR)	6.00 (4.00-6.50)	6.00 (4.50-7.00)	6.00 (4.00-6.50)	.02	5.46 (3.92-6.53)	5.67 (3.90-6.30)	0.0405
Time since MS onset, median (IQR), y	16.11 (9.67-23.97)	18.81 (11.28-27.23)	13.78 (8.83-21.75)	<.001	15.73 (8.68-23.72)	15.63 (9.33-23.69)	0.0157
Comorbidities							
Cardiovascular disease	38 (8.9)	22 (10.8)	16 (7.1)	.23	7.9	8.4	0.0053
Diabetes	21 (4.9)	10 (4.9)	11 (4.9)	>.99	4.6	5.5	0.0092
Obesity	31 (7.2)	15 (7.4)	16 (7.1)	>.99	6.7	7.0	0.0030
Pulmonary disease	18 (4.2)	12 (5.9)	6 (2.7)	.15	4.0	3.9	0.0011
Smoking	34 (7.9)	12 (5.9)	22 (9.7)	.20	7.3	7.8	0.0053
High-dose methylprednisolone during the month before COVID-19^c^	7 (1.6)	6 (3.0)	1 (0.4)	.06	1.6	1.4	0.0017
Vaccinated against COVID-19^d^	126 (29.4)	32 (15.8)	94 (41.6)	<.001	32.2	30.2	0.0200
SARS-CoV-2 variant^e^							
Original Alpha	263 (61.3)	152 (74.9)	111 (49.1)	<.001	59.5	61.0	0.0155
Delta	65 (15.2)	19 (9.4)	46 (20.4)		15.4	15.2	0.0023
Omicron	101 (23.5)	32 (15.8)	69 (30.5)		25.1	23.8	0.0132
Anti–COVID-19 monoclonal antibody treatment^f^	9 (2.1)	2 (1.0)	7 (3.1)	.18	1.6	2.1	0.0056
Anti-CD20 treatment							
Ocrelizumab	NA	NA	104 (46.0)	NA	NA	NA	NA
Rituximab	NA	NA	122 (54.0)	NA	NA	NA	NA
Cycles of anti-CD20 treatment, median (IQR), No.^g^	NA	NA	5.00 (3.00-8.00)	NA	NA	NA	NA
Time from last anti-CD20 cycle to COVID-19, median (IQR), mo^h^	NA	NA	3.91 (2.00-5.29)	NA	NA	NA	NA

Abbreviations: ASD, absolute standardized difference; EDSS, Expanded Disability Status Scale; MS, multiple sclerosis; NA, not applicable; PMS, progressive multiple sclerosis.

^a^ Data are presented as number (percentage) of patients, unless otherwise specified.

^b^ Data are presented as percentage of patients, unless otherwise specified.

^c^ A high dose was 500 mg/J or greater for at least 1 day.

^d^ Infection occurring at least 7 days after the second dose of COVID-19 vaccine.

^e^ The variant was considered dominant when it was present in more than 50% of the samples for a given week: original Alpha, before June 30, 2021; Delta, from June 30 to December 27, 2021; and Omicron, after December 27, 2021.

^f^ Treatment with COVID-19 monoclonal antibody in the 5 days after symptom onset.

^g^ Data were missing for 38 patients receiving anti-CD20 therapies.

^h^ Data were missing for 23 patients receiving anti-CD20 therapies.

### Association Between Anti-CD20 Therapies and Severe COVID-19 Among Patients With PMS

**Figure 2.**
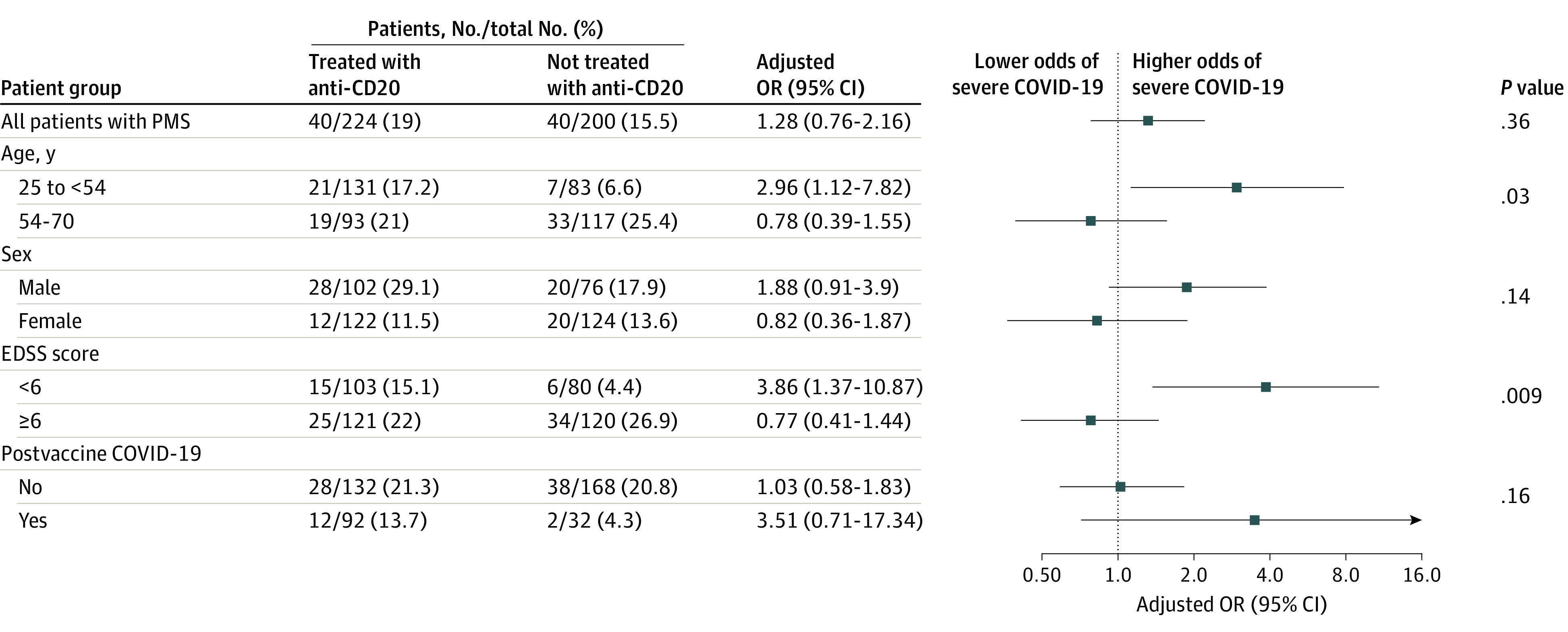
Association Between Anti-CD20 Therapies and Severe COVID-19 in All Patients With Progressive Multiple Sclerosis (PMS) and by Subgroup Observed numbers of events are reported in the nonweighted population without imputation of missing data. Percentages and odds ratios (ORs) were estimated in the weighted population with imputation of missing data (including outcomes). The analysis was propensity score weighted (average treatment effect estimand). Variables used in the propensity score estimation were age, sex, time since multiple sclerosis onset, Expanded Disability Status Scale (EDSS) score, comorbidities (cardiovascular disease, pulmonary disease, diabetes, obesity, and active tobacco smoking), exposure to high-dose methylprednisolone the month before COVID-19, postvaccine COVID-19 (infection occurring at least 7 days after the second dose of COVID-19 vaccine), COVID-19 variant (original Alpha, Delta, or Omicron), and treatment of COVID-19 with monoclonal antibody. Severe COVID-19 was defined as hospitalization with any mode of oxygenation or death. Age was divided into 2 groups based on the median for patients with PMS, and subgroup analyses were conducted in age categories defined accordingly. *P* values for subgroup analyses are for the interaction. Squares indicate ORs, with horizontal lines indicating 95% CIs.

Severe COVID-19 occurred in 40 patients treated with anti-CD20 (17.9%) and in 40 patients not treated with anti-CD20 (20.0%); anti-CD20 treatment was not associated with risk of severe COVID-19 in crude analysis (OR, 0.88; 95% CI, 0.54-1.43). In propensity score–weighted analysis, 19.0% of patients treated with anti-CD20 and 15.5% of those not treated with anti-CD20 had severe COVID-19, and anti-CD20 treatment was not associated with risk of severe COVID-19 (adjusted OR, 1.28; 95% CI, 0.76-2.16) (Figure 2). The subgroup analysis of patients with PMS showed a significant interaction between the presence of neurologic disability (EDSS score <6 or ≥6) and the association of anti-CD20 with severe COVID-19 (*P* = .009 for interaction). Anti-CD20 exposure among patients with PMS with an EDSS score of less than 6 was associated with increased risk of severe COVID-19 (adjusted OR, 3.86; 95% CI, 1.37-10.87), while no association was observed among patients with an EDSS score of 6 or higher (adjusted OR, 0.77; 95% CI, 0.41-1.44). There was also a negative interaction for age (*P* = .03 for interaction), with a higher risk of severe COVID-19 associated with anti-CD20 among patients younger than 54 years (adjusted OR, 2.96 [95% CI, 1.12-7.82]) and no association among patients aged 54 years or older (adjusted OR, 0.78; 95% CI, 0.39-1.55) (Figure 2 and eTable 2 in Supplement 1). Considering secondary outcomes, in weighted analysis, anti-CD20 therapies were not associated with any COVID-19 severity outcomes (eTable 3 and eFigure 2 in Supplement 1).

### Factors Associated With Severe COVID-19 Among Patients With PMS Treated With Anti-CD20

In the univariable analysis, among patients with nonsevere COVID-19 vs severe COVID-19, female sex (110 of 184 [59.8%] vs 12 of 40 [30.0%]) and the Omicron variant (63 [34.3%] vs 4 [10.0%]) were more frequent, while rituximab exposure was more frequent among patients with severe COVID-19 (28 of 40 [70.0%] vs 94 of 184 [51.1%]). Results are summarized in Table 3. In multivariable analysis, factors significantly associated with COVID-19 severity were female sex (OR, 0.29; 95% CI, 0.13-0.63) and the Omicron variant (OR, 0.24; 95% CI, 0.08-0.76).

**Table 3.** Factors Associated With Hospitalization With Oxygen or Higher Severity of COVID-19 Among Patients With PMS Receiving Anti-CD20 Therapies

Factor	Patients with PMS treated with anti-CD20^a^			Univariate analysis		Multivariate analysis	
	All (N = 226)	Without severe COVID-19 (n = 184)^b^	With severe COVID-19 (n = 40)^b^	OR (95% CI)	*P* value	OR (95% CI)	*P* value
Sex							
Female	124 (54.9)	110 (59.8)	12 (30.0)	0.29 (0.14-0.62)	.001	0.29 (0.13-0.63)	.002
Male	102 (45.1)	74 (40.2)	28 (70.0)	1 [Reference]		1 [Reference]	
Age, median (IQR), y	6.00 (4.00-6.50)	6.00 (4.00-6.50)	6.00 (5.50-6.50)	1.03 (0.99-1.08)	.14	1.04 (0.99-1.10)	.08
EDSS score, median (IQR)^c^	6.00 (4.00-6.50)	6.00 (4.00-6.50)	6.00 (5.50-6.50)	1.28 (0.98-1.66)	.07	1.27 (0.96-1.68)	.10
Time since MS onset, median (IQR), y	13.78 (8.83-21.75)	13.74 (8.72-21.39)	15.90 (9.67-24.00)	1.02 (0.98-1.06)	.32	NA	NA
Comorbidities							
Cardiovascular disease	16 (7.1)	13 (7.1)	3 (7.5)	1.07 (0.29-3.95)	.93	NA	NA
Diabetes	11 (4.9)	7 (3.8)	4 (10.0)	2.80 (0.77-10.15)	.12	NA	NA
Obesity	16 (7.1)	14 (7.6)	2 (5.0)	0.64 (0.14-2.95)	.56	NA	NA
Pulmonary disease	6 (2.7)	4 (2.2)	2 (5.0)	2.36 (0.41-13.51)	.33	NA	NA
Smoking	22 (9.7)	20 (10.9)	2 (5.0)	0.43 (0.10-1.94)	.27	NA	NA
Anti-CD20 treatment							
Ocrelizumab	104 (46.0)	90 (48.9)	12 (30.0)	1 [Reference]	.04	1 [Reference]	.32
Rituximab	122 (54.0)	94 (51.1)	28 (70.0)	2.20 (1.05-4.61)		1.49 (0.68-3.30)	
Cycles of anti-CD20 treatment, median (IQR), No.^d^	5.00 (3.00-8.00)	5.00 (3.00-8.00)	5.00 (4.00-7.00)	1.02 (0.90-1.16)	.77	NA	NA
Time from last anti-CD20 cycle to COVID-19, median (IQR), mo^e^	3.91 (2.00-5.29)	3.81 (2.10-5.45)	4.27 (2.00-5.26)	1.00 (0.94-1.07)	.98	NA	NA
High-dose methylprednisolone during the month before COVID-19^f^	1 (0.4)	1 (0.5)	0	NA	NA	NA	NA
Vaccinated against COVID-19^g^	94 (41.6)	80 (43.5)	12 (30.0)	0.56 (0.27-1.18)	.13	NA	NA
SARS-CoV-2 variant^h^							
Original Alpha	111 (49.1)	85 (46.2)	26 (65.0)	1 [Reference]	.02	1 [Reference]	.04
Delta	46 (20.4)	36 (19.6)	10 (25.0)	0.91 (0.40-2.09)		0.99 (0.41-2.39)	
Omicron	69 (30.5)	63 (34.2)	4 (10.0)	0.22 (0.07-0.67)		0.24 (0.08-0.76)	
Anti–COVID-19 monoclonal antibody treatment^i^	7 (3.1)	4 (2.2)	3 (7.5)	3.64 (0.78-17.10)	.10	NA	NA
Lymphocyte count, median (IQR), /μL^j^	1400 (1040-1820)	1380 (1017-1820)	1500 (1140-1975)	1.30 (0.64-2.66)	.47	NA	NA
IgG level, mean (SD), mg/dL^k^	925 (235)	922 (228)	942 (275)	0.95 (0.75-1.21)	.68	NA	NA

Abbreviations: EDSS, Expanded Disability Status Scale; IgG, immunoglobulin G; MS, multiple sclerosis; NA, not available; OR, odds ratio; PMS, progressive multiple sclerosis.

SI conversion factors: To convert IgG to g/L, multiply by 0.01; lymphocyte count to ×10^9^/L, multiply 0.001.

^a^ Data are presented as number (percentage) of patients, unless otherwise specified. Two patients (0.9%) had a nonavailable outcome.

^b^ Severe COVID-19 was indicated by a severity score of 4 or greater (ie, hospitalization with supplemental oxygen or higher severity).

^c^ Data were missing for 7 patients overall, 6 without severe COVID-19, and 1 with severe COVID-19.

^d^ Data were missing for 38 patients overall, 27 without severe COVID-19, and 11 with severe COVID-19.

^e^ Data were missing for 23 patients overall, 19 without severe COVID-19, and 3 with severe COVID-19; 1 had missing COVID-19 severity outcome.

^f^ A high dose was 500 mg/J or greater for at least 1 day.

^g^ Infection occurring at least 7 days after the second dose of COVID-19 vaccine.

^h^ The variant was considered dominant when it was present in more than 50% of the samples for a given week: original Alpha, before June 30, 2021; Delta, from June 30 to December 27, 2021; and Omicron, after December 27, 2021.

^i^ Treatment with COVID-19 monoclonal antibody in the 5 days after symptoms onset.

^j^ Data were missing for 82 patients overall, 64 without severe COVID-19, and 17 with severe COVID-19; 1 had missing COVID-19 severity outcome.

^k^ Data were missing for 94 patients overall, 72 without severe COVID-19, and 20 with severe COVID-19; 2 had missing COVID-19 severity outcome.

## Discussion

In this study, we found a differential association between anti-CD20 therapies and severe COVID-19 among patients with RRMS and PMS. Among patients with RRMS, anti-CD20 was associated with severe COVID-19, including in those who were vaccinated (although risk of severe COVID-19 was lower in vaccinated than in unvaccinated patients). Although risk of severe COVID-19 was higher in patients with PMS, there was no association between anti-CD20 exposure and COVID-19 severity. However, among patients with PMS who were younger and had less disability, anti-CD20 therapies were associated with higher risk of severe COVID-19. Female sex and the Omicron variant were associated with less-severe COVID-19 among anti-CD20–treated patients with PMS. Our study investigated a cohort of patients with MS and COVID-19. Therefore, it is not intended to estimate the risk of severe COVID-19 for all patients with RRMS or PMS, as this has been done in another study using a population-based approach.^15^

Among patients with RRMS, our results are consistent with other findings of an association between anti-CD20 therapies and an increased risk of severe COVID-19.^2,6,8^ Furthermore, this association was still observed even in fully vaccinated patients with RRMS. Anti-CD20 therapies have been shown to be associated with reduced effectiveness of vaccines both biologically^16,17^ and in terms of protection against COVID-19,^18^ including severe cases.^19^ B-cell counts have been identified as the most important factor associated with serological response after COVID-19 vaccination in patients with MS treated with rituximab.^20^ However, in our study, the absolute risk of severe COVID-19 was lower in vaccinated (7.0%) than unvaccinated (16.6%) patients with RRMS treated with anti-CD20, suggesting that vaccination should continue to be recommended for anti-CD20–treated patients with MS. Low mortality rates were observed (2 and 0 treated and untreated patients, respectively), likely due to the population’s young age, low cardiovascular risk, and limited neurologic disability.^21^ The type of anti-CD20 (rituximab or ocrelizumab) was not associated with COVID-19 severity among patients with RRMS treated with anti-CD20 in multivariate analysis. This result contrasts with previous findings.^4,8^ However, none of the previous analyses were stratified by MS course, and none were adjusted for the number of infusions, although that number was higher for patients receiving rituximab than those receiving ocrelizumab because ocrelizumab became available more recently in France.

By contrast, among patients with PMS, we observed a higher risk of severe COVID-19 than among patients with RRMS, but no association was found between anti-CD20 therapies and COVID-19 severity. Our study suggests that in patients with PMS, anti-CD20 therapies may be associated with COVID-19 severity among young patients and those without severe neurologic disability, while no association was found in older patients and those with more severe disability. These findings should be interpreted with caution, as they are the results of subgroup analyses. Neurologic disability and age are major factors associated with fatal COVID-19; in previous studies, reported COVID-19 deaths among patients with MS occurred almost exclusively in older, nonambulatory, and mostly untreated patients.^3,4,5^ Historical cohorts have shown that MS shortens life expectancy, with excess mortality associated with respiratory infections.^22,23^ A study showed that 75% of patients with MS who had an EDSS score of 7 or higher had chronic restrictive respiratory failure.^24^ However, the decline in life expectancy associated with MS has been more limited in recent years, potentially due to improved diagnosis and access to DMT.^22,25^ In the multivariable analysis of the factors associated with COVID-19 severity among anti-CD20–treated patients with PMS, 2 protective factors were identified: female sex and the Omicron variant. Both factors are also associated with less severe outcomes in the general population.^26^

### Strengths and Limitations

This study has several strengths. First, it is based on the large and well-documented multicenter COVISEP cohort study, which has a homogeneous national therapeutic strategy and health care access. Second, the study analyzed patients with RRMS and PMS separately, taking into account their different profiles. Third, the control groups used in the study were selected to focus on patients who could have received anti-CD20 therapies. Fourth, the propensity score–weighted analysis allowed us to estimate a marginal effect. Fifth, the main outcome chosen was COVID-19 severity, defined as hospitalization with supplemental oxygen, which is less sensitive to referral bias than hospitalization without supplemental oxygen and reflects objective respiratory insufficiency.

This study also has limitations. First, the COVISEP cohort study relies on voluntary reporting by treating neurologists, which may result in selection bias favoring the most severe cases. As the study was observational, some unmeasured confounding factors may persist, such as lack of information on past immunosuppression and the dose of rituximab at each infusion, which could also influence infection risk and severity. However, it is worth noting that the current practice in France is to administer 1000 mg of rituximab per infusion. We also did not correct for multiple analyses; thus, secondary analyses should be viewed with caution. Since certain variables contained missing values, we conducted multiple imputations assuming that the missing data were rare and missing at random. Our study may have had limited statistical power in certain subgroups. For instance, the absence of an association between anti-CD20 therapies and COVID-19 severity in patients with PMS does not imply that anti-CD20 therapy has no consequences in this population, which is already at high risk for severe infections. Finally, our study did not investigate the association of anti-CD20 therapies with long-term outcomes of COVID-19 among patients with MS.

## Conclusions

In this cohort study of patients with MS and COVID-19, among patients not treated with anti-CD20 therapies, those with PMS had a higher risk of severe COVID-19 compared with those with RRMS. In patients with RRMS, anti-CD20 therapies were associated with an increased risk of severe COVID-19. There was no association between treatment with anti-CD20 therapy and risk of severe COVID-19 in patients with PMS.

## Notes

Supplement 1. **eMethods 1.** Details of Included Data **eMethods 2.** Description of Multiple Imputation Method **eTable 1.** Demographic and Clinical Characteristics and Outcomes of Patients With Recurrent-Remittent and Progressive MS Treated With Ocrelizumab and Rituximab **eTable 2.** Subgroup Analysis of the Association of Anti-CD20 and Severe COVID-19 in Patients With Recurrent-Remittent and Progressive MS, Unadjusted and Propensity Weighted Analysis **eTable 3.** Association of Anti-CD20 Exposure and COVID-19 Outcomes in Patients With Recurrent-Remittent and Progressive MS, Unadjusted Analysis and Propensity Weighted Analysis **eTable 4.** Factors Associated With Hospitalization With Oxygen or Higher Severity Among Patients With Recurrent-Remittent MS Treated with Anti-CD20 Therapies **eFigure 1.** Flow Chart **eFigure 2.** Association Between Anti-CD20 Exposure and COVID-19 Outcomes Among Patients With RRMS and PMS

Supplement 2.
Data Sharing Statement
