# Supplementary material for: Association Between Anti-CD20 Therapies and COVID-19 Severity Among Patients With Relapsing-Remitting and Progressive Multiple Sclerosis
Source: JAMA Netw Open. 2023 Jun 23;6(6):e2319766. doi: 10.1001/jamanetworkopen.2023.19766 (PMC10290250; doi:10.1001/jamanetworkopen.2023.19766)
Supplement: Supplement 1. — eMethods 1. Details of Included Data eMethods 2. Description of Multiple Imputation Method eTable 1. Demographic and Clinical Characteristics and Outcomes of Patients With Recurrent-Remittent and Progressive MS Treated With Ocrelizumab and Rituximab eTable 2. Subgroup Analysis of the Association of Anti-CD20 and Severe COVID-19 in Patients With Recurrent-Remittent and Progressive MS, Unadjusted and Propensity Weighted Analysis eTable 3. Association of Anti-CD20 Exposure and COVID-19 Outcomes in Patients With Recurrent-Remittent and Progressive MS, Unadjusted Analysis and Propensity Weighted Analysis eTable 4. Factors Associated With Hospitalization With Oxygen or Higher Severity Among Patients With Recurrent-Remittent MS Treated with Anti-CD20 Therapies eFigure 1. Flow Chart eFigure 2. Association Between Anti-CD20 Exposure and COVID-19 Outcomes Among Patients With RRMS and PMS [file jamanetwopen-e2319766-s001.pdf]

Supplementary Online Content

Januel E, Hajage D, Labauge P, et al. Association between anti-CD20 therapies and COVID-19 severity among patients with relapsing-remitting and progressive multiple sclerosis. *JAMA Netw Open*. 2023;6(6):e2319766. doi:10.1001/jamanetworkopen.2023.19766

eMethods 1. Details of Included Data

eMethods 2. Description of Multiple Imputation Method

eTable 1. Demographic and Clinical Characteristics and Outcomes of Patients With Recurrent-Remittent and Progressive MS Treated With Ocrelizumab and Rituximab

eTable 2. Subgroup Analysis of the Association of Anti-CD20 and Severe COVID-19 in Patients With Recurrent-Remittent and Progressive MS, Unadjusted and Propensity Weighted Analysis

eTable 3. Association of Anti-CD20 Exposure and COVID-19 Outcomes in Patients With Recurrent-Remittent and Progressive MS, Unadjusted Analysis and Propensity Weighted Analysis

eTable 4. Factors Associated With Hospitalization With Oxygen or Higher Severity Among Patients With Recurrent-Remittent MS Treated with Anti-CD20 Therapies

eFigure 1. Flow Chart

eFigure 2. Association Between Anti-CD20 Exposure and COVID-19 Outcomes Among Patients With RRMS and PMS

This supplementary material has been provided by the authors to give readers additional information about their work.

© 2023 Januel E et al. *JAMA Network Open*.

E-Method 1: **Detail of included data**

For all patients were recorded: age, gender, date of MS diagnosis, type of MS (RR-MS or P-MS), EDSS score (15) before COVID-19, current MS DMT, comorbidities (cardiovascular disease, pulmonary disease, diabetes, obesity [BMI > 30 kg/m2], active tobacco smoking), exposure to high dose methylprednisolone during the last month before COVID-19 (methylprednisolone ≥ 500 mg / J ≥ 1 day), vaccination before COVID-19 (defined as an infection occurring at least 7 days after the second dose of COVID-19 vaccine). Regarding COVID-19, were collected: date of symptoms onset, type and date of COVID-19 confirmatory testing positivity (PCR, antigen test), treatment with COVID-19 monoclonal antibody in the 5 days after symptoms onset. COVID-19 severity was defined at the nadir state, according to an ordinal scale ranging from 1 to 7: 1.No limitations on activities; 2.Not hospitalized, limitation on activities; 3.Hospitalized, not requiring supplemental oxygen; 4. Hospitalized, requiring supplemental oxygen; 5.Hospitalized, on non-invasive ventilation or high flow oxygen devices; 6.Hospitalized, on invasive mechanical ventilation; 7.Death. COVID-19 variant was approximated at the date of infection (symptoms onset or if not available date of confirmatory testing positivity) from the weekly French COVID-19 variant surveillance: the proxy for the variant was the one considered dominant at the date of infection, i.e. representing more than 50% of the positive samples in the week of interest: “Original-Alpha” before 30/06/2021, “Delta”: 30/06/2021-27/12/2021, and “Omicron”: after 27/12/2021. For anti-CD20 treated patients, number of anti-CD20 infusions, lymphocyte count and immunoglobulin G, were also recorded. Data were recorded by the treating neurologist at the subsequent consultation following COVID-19 resolution, or at the time of COVID-19-related-hospitalization for the most severe patients.

© 2023 Januel E et al. *JAMA Network Open*.

E-Method 2: Description of multiple imputation method

1) **Multiple imputation for analysis of the impact of anti-CD20 therapies on COVID-19 severity:**

Multiple imputation with the chained equations (MICE) technique was used to replace missing values when appropriate, assuming that missing data were missing at random:

For RR-MS patients on the one hand and P-MS patients in the other, ten copies of the datasets were created separately in anti-CD20 treated and non-treated groups, with the missing values replaced by imputed values.

Variables used in the imputation phase

The following variables were used in the imputation phase:

- age, gender, time from MS diagnosis, EDSS score before COVID-19, presence of cardiovascular disease, pulmonary disease, diabetes, obesity, active tobacco smoking), exposure to high dose methylprednisolone during the last month before COVID-19, post vaccine COVID-19, date of COVID-19 symptoms onset, type and date of COVID-19 confirmatory testing positivity (PCR, antigen test), treatment with COVID-19 monoclonal antibody in the 5 days after symptoms onset. COVID-19 severity at the nadir state, COVID-19 variant

Procedures chosen and software used

Multiple-imputation analyses using chained equations procedure, with linear imputation for continuous variables, proportional odds regression for ordinal variables, and logistic or multinomial regression for categorical variables were used. Analyses were performed using the R software and the mice package.

© 2023 Januel E et al. *JAMA Network Open*.

Number of imputations: 10

Pooling procedures for analysis :

Results from each imputed dataset were pooled using Rubin's rules to obtain overall estimates and their standard errors.

2) Multiple imputation for the analysis of the risk factors for COVID-19 severity among anti-CD20 treated patients

Some variables were recorded only for anti-CD20 treated patients, such as immunoglobulin G level, lymphocytes count level, number of cycles of antiCD20 treatment, delay between last anti-CD20 cycle and COVID-19. For all anti-CD20 treated patients, ten copies of the datasets were imputed with the missing values replaced by imputed values.

Variables used in the imputation phase

The following variables were used in the imputation phase: age, gender, time from MS diagnosis, EDSS score before COVID-19, presence of cardiovascular disease, pulmonary disease, diabetes, obesity, active tobacco smoking), exposure to high dose methylprednisolone during the last month before COVID-19, post vaccine COVID-19, date of COVID-19 symptoms onset, type and date of COVID-19 confirmatory testing positivity (PCR, antigen test), treatment with COVID-19 monoclonal antibody in the 5 days after symptoms onset. COVID-19 severity at the nadir state, COVID-19 variant, lymphocytes count level, immunoglobulin G level, number of cycles of antiCD20 treatment, delay between last anti-CD20 cycle and COVID-19

Procedures chosen and software used

© 2023 Januel E et al. *JAMA Network Open*.

Multiple-imputation analyses using chained equations procedure, with linear imputation for continuous variables, proportional odds regression for ordinal variables, and logistic or multinomial regression for categorical variables were used. Analyses were performed using the R software and the mice package.

Number of imputations: 10

Pooling procedures for analysis

Results from each imputed dataset were pooled using Rubin's rules to obtain overall estimates and their standard errors.

© 2023 Januel E et al. *JAMA Network Open*.

e-Table 1: Demographic, clinical characteristics and outcomes of ocrelizumab and rituximab treated recurrent-remittent and progressive MS patients

Anti-CD20 treated Relapsing Remitting Multiple Sclerosis Patients				
	Overall	RR-MS treated with ocrelizumab	RR-MS treated with rituximab	p
N	418	350 (83.7)	68 (16.3)	
Gender				0.59
- Males	91 (21.8) (1)	74 (21.2) (1)	17 (25.0) (0)	
- Female	326 (78.2) (1)	275 (78.8) (1)	51 (75.0) (0)	
Age, years	39.15 [31.22, 45.81] (3)	38.67 [31.09, 45.43] (3)	42.46 [34.30, 48.55] (0)	0.05
EDSS	2.00 [1.00, 4.00] (26)	2.00 [1.00, 4.00] (25)	3.00 [1.50, 4.25] (1)	0.02
Delay from MS onset, years	8.96 [4.70, 14.78] (11)	8.61 [4.27, 14.19] (7)	10.94 [7.26, 15.96] (4)	0.02
Comorbidities				
- Cardiovascular disease	11 (2.6)	11 (3.1)	0 (0.0)	0.22
- Diabetes	7 (1.7)	5 (1.4)	2 (2.9)	0.32
- Obesity	19 (4.5)	13 (3.7)	6 (8.8)	0.13
- Pulmonary disease	12 (2.9)	8 (2.3)	4 (5.9)	0.11
- Smoking	42 (10.0)	36 (10.3)	6 (8.8)	0.88
Number of cycles of antiCD20 treatment	4.00 [2.00, 5.00] (61)	3.00 [2.00, 5.00] (49)	5.00 [4.00, 8.00] (12)	<0.001
Delay between last anti-CD20 cycle and COVID-19, months	3.35 [1.54, 5.03] (30)	3.29 [1.48, 4.77] (22)	4.90 [2.07, 10.85] (8)	0.001
High dose methylprednisolone during the month before COVID-19	4 (1.0) (1)	4 (1.1) (1)	0 (0.0) (0)	1.00
Vaccinated against COVID-19	169 (40.4)	144 (41.1)	25 (36.8)	0.59
Sars-COV-2 variant				0.04
- Original-alpha	211 (50.5)	167 (47.7)	44 (64.7)	
- delta	70 (16.7)	61 (17.4)	9 (13.2)	
- omicron	137 (32.8)	122 (34.9)	15 (22.1)	
Anti-COVID-19 Monoclonal antibody treatment	17 (4.1)	16 (4.6)	1 (1.5)	0.33
Lymphocyte count/mm3	1500 [1150, 1955] (132)	1495 [1123, 1896] (114)	1516 [1230, 2105] (18)	0.36
Immunoglobulin G, mg/dL, mean (SD) (NA)	930 (244) (142)	951 (242) (114)	837 (2.30) (16)	0.002
Outcome				

© 2023 Januel E et al. *JAMA Network Open*.

- Hospitalization or higher severity	61 (15.2)	44 (13.0)	17 (27.0)	0.008
- Hospitalization with oxygen or higher severity	51 (12.7)	36 (10.7)	15 (23.8)	0.008
- Non-invasive or invasive ventilation or higher severity	19 (4.7)	12 (3.6)	7 (11.1)	0.02
- Death	2 (0.5)	1 (0.3)	1 (1.6)	0.72
- NA	(17)	(12)	(5)	

Anti-CD20 treated Progressive Multiple Sclerosis Patients

	Overall	P-MS treated with ocrelizumab	P-MS treated with rituximab	p
N	226	104 (46.0)	122 (54.0)	
Gender				0.15
- Males	102 (45.1)	41 (39.4)	61 (50.0)	
- Female	124 (54.9)	63 (60.6)	61 (50.0)	
Age, years	53.16 [46.84, 58.62]	52.98 [46.45, 59.41]	53.34 [47.75, 57.41]	0.82
EDSS	6.00 [4.00, 6.50]	5.50 [4.00, 6.50]	6.00 [4.12, 6.50]	0.13
Delay from MS onset	13.78 [8.83, 21.75]	14.10 [9.41, 21.57]	13.67 [8.63, 22.16]	0.54
MS course				0.09
- Primary progressive	95 (42.0)	37 (35.6)	58 (47.5)	
- Secondary progressive	131 (58.0)	67 (64.4)	64 (52.5)	
Comorbidities				
- Cardiovascular disease	16 (7.1)	6 (5.8)	10 (8.2)	0.61
- Diabetes	11 (4.9)	5 (4.8)	6 (4.9)	1.00
- Obesity	16 (7.1)	4 (3.8)	12 (9.8)	0.14
- Pulmonary disease	6 (2.7)	1 (1.0)	5 (4.1)	0.22
- Smoking	22 (9.7)	10 (9.6)	12 (9.8)	1.00
Number of cycles of antiCD20 treatment	5.00 [3.00, 8.00] (38)	4.00 [3.00, 6.00] (22)	6.00 [4.00, 8.00] (16)	0.004
Delay between last anti-CD20 cycle and COVID-19	3.91 [2.00, 5.29] (23)	3.86 [1.81, 5.14] (12)	3.94 [2.28, 5.42] (11)	0.43
High dose methylprednisolone during the month before COVID-19,	1 (0.4)	0 (0.0)	1 (0.8)	1.00
Vaccinated against COVID-19	94 (41.6)	50 (48.1)	44 (36.1)	0.09
Sars-COV-2 variant				0.002
- Original-alpha	111 (49.1)	42 (40.4)	69 (56.6)	

© 2023 Januel E et al. *JAMA Network Open*.

- delta	46 (20.4)	18 (17.3)	28 (23.0)	
- omicron	69 (30.5)	44 (42.3)	25 (20.5)	
Anti-COVID-19 Monoclonal antibody treatment	7 (3.1)	2 (1.9)	5 (4.1)	0.46
Lymphocyte count/mm3	1400 [1040, 1820]	1530 [1074, 1955]	1389 [987, 1626]	0.12
Immunoglobulin G, mg/dL, mean (SD) (NA)	925 (235) (94)	934 (212) (58)	920 (247) (36)	0.74
Outcome				
- Hospitalization or higher severity	56 (25.0)	16 (15.7)	40 (32.8)	0.005
- Hospitalization with oxygen or higher severity	40 (17.9)	12 (11.8)	28 (23.0)	0.05
- Non-invasive or invasive ventilation or higher severity	23 (10.3)	5 (4.9)	18 (14.8)	0.03
- Death	3 (1.3)	0 (0.0)	3 (2.5)	0.31
- NA	(2)	(2)	(0)	

Data are N (%) / median (P25; P75) and (NA:number non available), unless otherwise specified

© 2023 Januel E et al. *JAMA Network Open*.

e-table 2: Subgroup analysis of the association of anti-CD20 and severe COVID-19 in recurrent-remittent and progressive MS patients, unadjusted and propensity weighted analysis

	Included Remittent Recurrent Multiple Sclerosis Patients							
	Unadjusted analysis				Propensity Weighted analysis			
	Not treated with anti-CD20, N (%)	Treated with anti-CD20, N (%)	OR (95% CI)	P value for interaction	Not treated with anti-CD20, %	Treated with anti-CD20, %	Adjusted-OR (95% CI)	P value for interaction
Age (years)				0.64				0.94
[17;39[	4/260 (1.5)	19/200 (9.5%)	6.68 (2.12; 21.1)		1.9%	9.3%	5.42 (1.73;17)	
[39-75[	10/262 (3.8)	32/198 (16.2%)	4.84 (2.33;10.06)		3.9%	17.4%	5.12 (2.38;10.99)	
Gender				0.94				0.68
Males	3/132 (2.3)	9/90 (10.0)	5.03 (1.31;19.23)		2.3%	13.4%	6.73 (1.66;27.3)	
Females	11/391 (2.8)	42/310 (13.5)	5.33 (2.69;10.57)		3.1%	13.4%	4.85 (2.43;9.69)	
EDSS				0.34				0.59
<6	12/483 (2.5)	43/343 (12.5)	13.16 (1.44;120.46)		2.6%	12.8%	5.5 (2.8;10.82)	
>=6	2/23 (8.7)	8/38 (21.1)	2.30 (0.48;11.07)		7.1%	20.5%	3.31 (0.60;18.34)	
Post-vaccine				0.63				0.57

© 2023 Januel E et al. *JAMA Network Open*.

No	13/384 (3.4)	40/242 (16.5)	5.65 (2.94; 10.89)		3.9%	16.6%	4.87 (2.48;9.59)	
Yes	1/140 (0.7)	11/159 (6.9)	9.38 (1.29;68.04)		0.9%	7%	8.85 (1.26;62.12)	

Included Progressive Multiple Sclerosis Patients

	Unadjusted analysis				Propensity Weighted analysis			
Severity	Not treated with anti-CD20, N (%)	Treated with anti-CD20, N (%)	OR (95% CI)	P value	Not treated with anti-CD20, (%)	Treated with anti-CD20, (%)	Adjusted-OR (95% CI)	P value for interaction
Age (years)				0.05				0.03
[25;54[	7/83 (8.4)	21/131 (16.0)	2.07 (0.84;5.13)		6.6%	17.2%	2.96 (1.12;7.82)	
[54-70[	33/117 (28.2)	19/93 (20.4)	0.68 (0.35;1.30)		25.4%	21%	0.78 (0.39;1.55)	
Gender				0.26				0.14
Males	20/76 (26.3)	28/102 (27.5)	1.07 (0.55;2.10)		17.9%	29.1%	1.88 (0.91;3.9)	
Females	20/124 (16.1)	12/122 (9.8)	0.59 (0.28;1.28)		13.6%	11.5%	0.82 (0.36;1.87)	
EDSS				0.06				0.009
<6	6/80 (7.5)	15/103 (14.6)	4.96 (0.80;30.6)		4.4%	15.1%	3.86 (1.37;10.87)	
>=6	34/120 (28.3)	25/121 (20.7)	0.33 (0.10;1.13)		26.9%	22%	0.77 (0.41;1.44)	
Post-vaccine				0.28				0.16
No	38/168 (22.6)	28/132 (21.2)	0.93 (0.54;1.62)		20.8%	21.3%	1.03 (0.58;1.83)	

© 2023 Januel E et al. *JAMA Network Open*.

Yes	2/32 (6.2)	12/92 (13.0)	2.32 (0.49;11.05)	4.3%	13.7%	3.51 (0.71;17.34)

Propensity-weighted analysis (ATE estimand). Variables used in the propensity score estimation: age, gender, delay from MS onset, EDSS score, comorbidities (cardiovascular disease, pulmonary disease, diabetes, obesity, active tobacco smoking), exposure to high dose methylprednisolone the month before COVID-19, post-vaccine COVID-19, COVID-19 variant (Original-Alpha, Delta or Omicron), and treatment of COVID-19 with monoclonal antibody

© 2023 Januel E et al. *JAMA Network Open*.

e-table 3: Association of anti-CD20 exposure and COVID-19 outcomes in recurrent-remittent and progressive MS patients, unadjusted analysis and propensity weighted analysis

Included Remittent Recurrent Multiple Sclerosis Patients								
	Unadjusted analysis				Propensity Weighted analysis			
Severity	Not treated with anti-CD20, N (%)	Treated with anti-CD20, N (%)	OR (95% CI)	P value	Not treated with anti-CD20, %	Treated with anti-CD20, %	Adjusted-OR (95% CI)	P value
>= Hospitalization	18 (3.4%)	61 (15.2%)	5.01 (2.92;8.57)	<0.001	3.7%	16.0%	4.92 (2.83;8.56)	<0.001
>= Hospitalization with O2	14 (2.7%)	51 (12.7%)	5.32 (2.89;9.81)	<0.001	2.9%	13.4%	5.2 (2.78;9.71)	<0.001
>= Hospitalization with noninvasive ventilation or ICU	4 (0.8%)	19 (4.7%)	6.23 (2.11;18.38)	0.001	0.8%	4.5%	5.72 (1.92;17.02)	0.002
Death	0 (0.0%)	2 (0.5%)	-	-	0.0%	0.4%	-	-
Included Progressive Multiple Sclerosis Patients								
	Unadjusted analysis				Propensity Weighted analysis			

© 2023 Januel E et al. *JAMA Network Open*.

Severity	Not treated with anti-CD20, N (%)	Treated with anti-CD20, N (%)	OR (95% CI)	P value	Not treated with anti-CD20, (%)	Treated with anti-CD20, (%)	Adjusted-OR (95% CI)	P value
>= Hospitalization	50 (25.0%)	56 (25.0%)	1.01 (0.65;1.57)	0.97	19.5%	26.3%	1.47 (0.91;2.38)	0.11
>= Hospitalization with O2	40 (20.0%)	40 (17.9%)	0.88 (0.54;1.43)	0.59	15.5%	19%	1.28 (0.76;2.16)	0.36
>= Hospitalization with noninvasive ventilation or ICU	22 (11.0%)	23 (10.3%)	0.94 (0.5;1.75)	0.81	8.9%	10.9%	1.25 (0.65;2.42)	0.51
Death	5 (2.5%)	3 (1.3%)	0.55 (0.13;2.34)	0.39	1.8%	1.5%	0.83 (0.19;3.63)	0.80

Propensity-weighted analysis (ATE estimand). Variables used in the propensity score estimation: age, gender, delay from MS onset, EDSS score, comorbidities (cardiovascular disease, pulmonary disease, diabetes, obesity, active tobacco smoking), exposure to high dose methylprednisolone the month before COVID-19, post-vaccine COVID-19, COVID-19 variant (Original-Alpha, Delta or Omicron), and treatment of COVID-19 with monoclonal antibody

© 2023 Januel E et al. *JAMA Network Open*.

E-table 4: Risk factors for hospitalization with oxygen or higher severity among recurrent-remittent-MS patients treated with anti-CD20 therapies

	All anti-CD20 RR-MS patients (N=418)	Anti-CD20 RR-MS patients without severe COVID-19 (N=350)	Anti-CD20 RR-MS patients with severe COVID-19 (N=51)	Univariate OR (IC95%)	P-value	Multivariate OR (IC95%)	P-alue
N (%) *	418	350 (87.3)	51 (12.7)				
Gender							
- Male	91 (21.8) (1)	81 (23.2) (1)	9 (17.6) (0)	1 (ref)			
- Female	326 (78.2) (1)	268 (76.8) (1)	42 (82.4) (0)	1.37 (0.64;2.92)	0.42		
Age, years	39.15 (31.22, 45.81) (3)	38.61 (31.06, 44.80) (3)	45.68 (32.49, 49.95) (0)	1.04 (1.01;1.07)	0.009	1.03 (0.99;1.06)	0.11
EDSS	2.00 (1.00, 4.00) (26)	2.00 (1.00, 3.50) (20)	3.00 (2.00, 4.25) (0)	1.22 (1.05;1.41)	0.01	1.11 (0.94;1.32)	0.21
Delay from MS onset, years	8.96 (4.70, 14.78) (11)	8.87 (4.44, 14.67) (9)	11.84 (6.05, 15.36) (1)	1.03 (0.99;1.07)	0.10		
Comorbidities							
- Cardiovascular disease	11 (2.6)	9 (2.6)	2 (3.9)	1.51 (0.32;7.22)	0.61		
- Diabetes	7 (1.7)	6 (1.7)	1 (2.0)	1.12 (0.13;9.55)	0.92		
- Obesity	19 (4.5)	17 (4.9)	2 (3.9)	0.78 (0.17;3.5)	0.75		
- Pulmonary disease	12 (2.9)	10 (2.9)	1 (2.0)	0.71 (0.09;5.83)	0.75		
- Smoking	42 (10.0)	38 (10.9)	4 (7.8)	0.68 (0.23;2)	0.49		
Anti-CD20 treatment							
- Ocrelizumab	350 (83.7)	302 (86.3)	36 (70.6)	1 (ref)		1 (ref)	
- Rituximab	68 (16.3)	48 (13.7)	15 (29.4)	2.59 (1.29;5.19)	0.008	1.72 (0.8;3.74)	0.17
Number of cycles of antiCD20 treatment	4.00 (2.00, 5.00) (61)	3.00 (2.00, 5.00) (51)	4.50 (4.00, 6.00) (9)	1.15 (1.01;1.32)	0.04	1.14 (0.98;1.34)	0.09
Delay between last anti-CD20 cycle and COVID-19, months	3.35 (1.54, 5.03) (30)	3.61 (1.51, 5.16) (25)	2.64 (1.58, 4.07) (3)	0.94 (0.84;1.04)	0.23		

© 2023 Januel E et al. *JAMA Network Open*.

High dose methylprednisolone during the month before COVID-19	4 (1.0) (1)	4 (1.1) (1)	0 (0.0) (0)				
Vaccinated against COVID-19	169 (40.4)	148 (42.3)	11 (21.6)	0.4 (0.2;0.79)	0.009	0.41 (0.14;1.16)	0.09
Sars-COV-2 variant					0.01		0.06
- Original-alpha	211 (50.5)	171 (48.9)	34 (66.7)	1 (ref)		1 (ref)	
- delta	70 (16.7)	55 (15.7)	11 (21.6)	1 (0.47;2.11)		1.82 (0.67;4.99)	
- omicron	137 (32.8)	124 (35.4)	6 (11.8)	0.26 (0.11;0.65)		0.52 (0.15;1.74)	
Anti-COVID-19 Monoclonal antibody treatment	17 (4.1)	14 (4.0)	2 (3.9)	0.89 (0.2;4.03)	0.88		
Lymphocyte count/mm3	1500 (1150, 1955) (132)	1500 (1142, 1933) (112)	1480 (1104, 1677) (17)	0.94 (0.62;1.42)	0.77		
Immunoglobulin G, mg/dL, mean (SD) (NA)	930 (244) (142)	938 (240) (121)	847 (258) (16)	0.88 (0.76;1.02)	0.10		

*17 patients (4.1%) with non-available outcome

Data are N (%) / median (P25; P75) and (NA: number non available), unless otherwise specified

RR-MS: relapsing remitting multiple sclerosis. Severe COVID-19: severity score ≥ 4, i.e. hospitalization with oxygen, or higher severity. High dose methylprednisolone: methylprednisolone >= 500 mg / J for at least one day; Complete vaccination: infection occurring at least 7 days after the second dose of COVID-19 vaccine; Sars-COV-2 variant: the variant was considered dominant when it was present in more than 50% of the samples for a given week : “Original-Alpha”: before 30/06/2021, “Delta”: from 30/06/2021 to 27/12/2021, and “Omicron”: after 27/12/2021; Anti-COVID-19 Monoclonal antibody treatment: treatment with COVID-19 monoclonal antibody in the 5 days after symptoms onset.

© 2023 Januel E et al. *JAMA Network Open*.

e-figure1: Flow chart

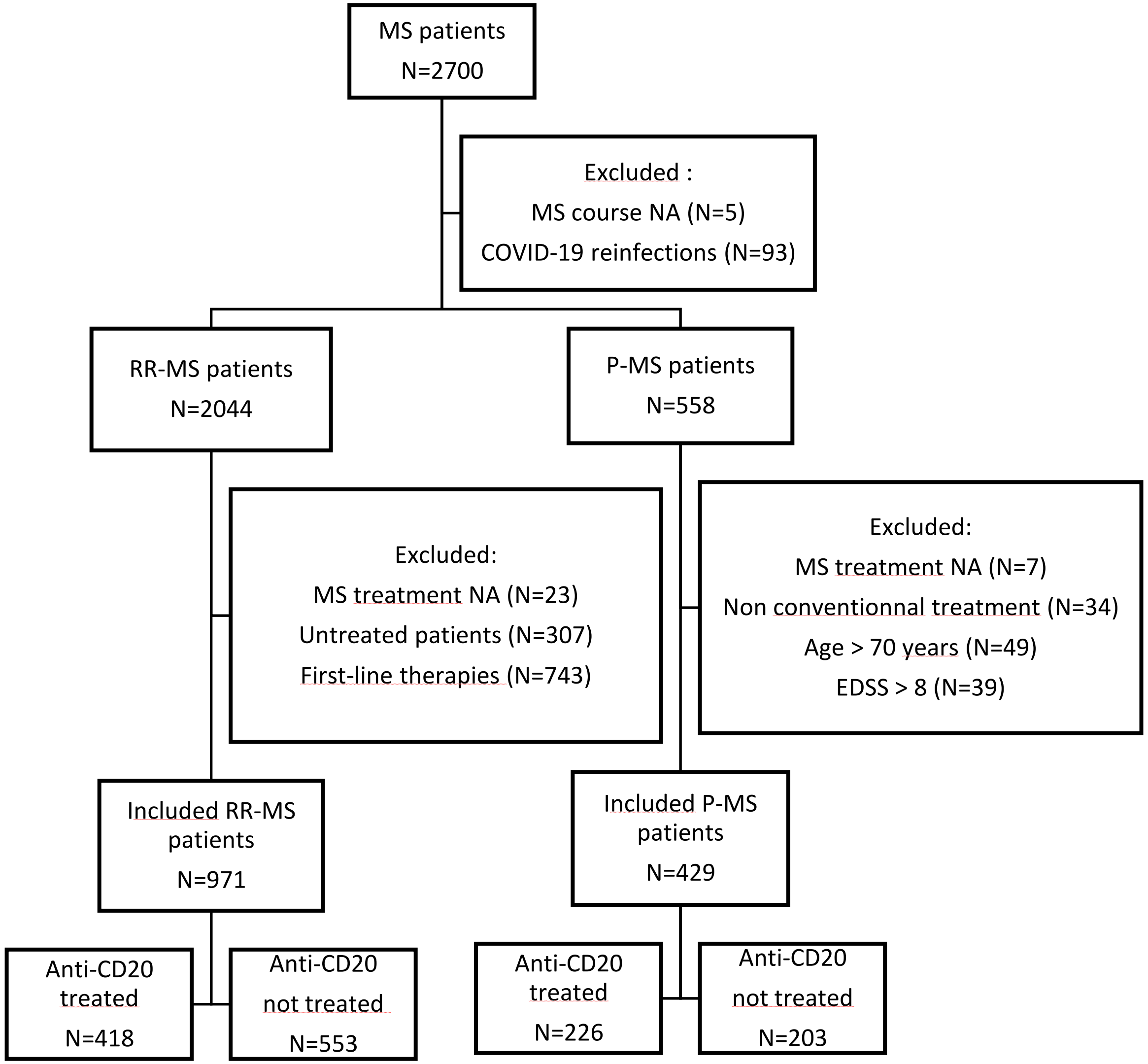

Excluded non-conventional treatments for P-MS patients: cyclophosphamide, alemtuzumab, mycophenolate mofetil, azathioprine, methotrexate; NA: non-available

© 2023 Januel E et al. *JAMA Network Open*.

e-figure 2: **Association between anti-CD20 exposure and COVID-19 outcomes (COVID-19 severity score ≥ hospitalization; ≥5; ≥hospitalization with O2; ≥ Non-invasive ventilation or ICU; death) among RR-MS and P-MS**

Impact of anti-CD20 exposure on the risk of severe COVID-19 among RR-MS and P-MS

	Treated with anti-CD20 N/tot (%)*	Not treated with anti-CD20 N/tot (%)*	Adjusted OR (95%CI)	
RR-MS patients				
>= Hospitalisation	61/401 (16%)	18/524 (3.7%)	4.92 (2.83;8.56)	<0.001
>= Hospitalisation with O2	51/401 (13.4%)	14/524 (2.9%)	5.2 (2.78;9.71)	<0.001
>= Noninvasive ventilation-ICU	19/401 (4.5%)	4/524 (0.8%)	5.72 (1.92;17.02)	0.002
Death	2/401 (0.5%)	0/524 (0%)		
P-MS patients				
>= Hospitalisation	56/224 (26.3%)	50/200 (19.5%)	1.47 (0.91;2.38)	0.112
>= Hospitalisation with O2	40/224 (19%)	40/200 (15.5%)	1.28 (0.76;2.16)	0.361
>= Noninvasive ventilation-ICU	23/224 (10.9%)	22/200 (8.9%)	1.25 (0.65;2.42)	0.506
Death	3/224 (1.5%)	5/200 (1.8%)	0.83 (0.19;3.63)	0.801

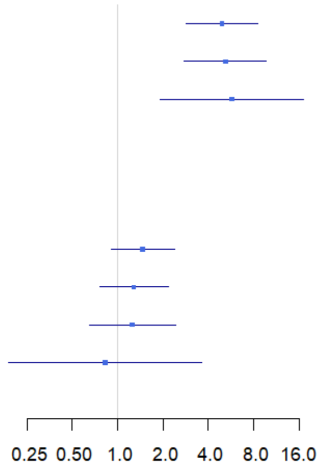

*Observed numbers of events are reported in the non-weighted population without imputation of missing data. Percentages and odd ratios are estimated on the weighted population with imputation of missing data (including outcomes). Propensity-weighted analysis (ATE estimand). Variables used in the propensity score estimation: age, gender, delay from MS onset, EDSS score, comorbidities (cardiovascular disease, pulmonary disease, diabetes, obesity, active tobacco smoking), exposure to high dose methylprednisolone the month before COVID-19, post-vaccine COVID-19, COVID-19 variant (Original-Alpha, Delta or Omicron), and treatment of COVID-19 with monoclonal antibody

ICU: intensive care unit

© 2023 Januel E et al. *JAMA Network Open*.
