# Supplementary material for: Association Between Anti-CD20 Therapies and COVID-19 Severity Among Patients With Relapsing-Remitting and Progressive Multiple Sclerosis
Source: JAMA Netw Open. 2023 Jun 23;6(6):e2319766. doi: 10.1001/jamanetworkopen.2023.19766 (PMC10290250; doi:10.1001/jamanetworkopen.2023.19766)
Supplement: Supplement 2. — Data Sharing Statement [file jamanetwopen-e2319766-s002.pdf]

Data Sharing Statement

Januel. Association Between Anti-CD20 Therapies and COVID-19 Severity Among Patients With Relapsing-Remitting and Progressive Multiple Sclerosis. *JAMA Netw Open*. Published June 23, 2023. doi:10.1001/jamanetworkopen.2023.19766

Data
Data available: No

Additional Information
Explanation for why data not available: Requests for access to the data reported in this paper will be considered by the corresponding author.
